# Multiple Endocrine Neoplasia Type 1 (MEN1) Phenocopy Due to a Cell Cycle Division 73 (*CDC73*) Variant

**DOI:** 10.1210/jendso/bvaa142

**Published:** 2020-09-26

**Authors:** Kate E Lines, Lisa B Nachtigall, Laura E Dichtel, Treena Cranston, Hannah Boon, Xun Zhang, Kreepa G Kooblall, Mark Stevenson, Rajesh V Thakker

**Affiliations:** 1 Academic Endocrine Unit, OCDEM, Radcliffe Department of Medicine, University of Oxford, Churchill Hospital, Oxford, UK; 2 Neuroendocrine Unit, Massachusetts General Hospital/Harvard Medical School, Boston, Massachusetts

**Keywords:** pancreatic neuroendocrine neoplasm, primary hyperparathyroidism, acromegaly, RNA-Scope

## Abstract

Multiple endocrine neoplasia type 1 (MEN1) is an autosomal dominant disorder characterized by the combined occurrence of parathyroid tumors, pituitary adenomas, and pancreatic neuroendocrine neoplasms (PNENs). MEN1 is caused by germline *MEN1* mutations in > 75% of patients, and the remaining 25% of patients may have mutations in unidentified genes or represent phenocopies with mutations in genes such as cell cycle division 73 (*CDC73)*, the calcium sensing receptor (*CASR)*, and cyclin-dependent kinase inhibitor 1B (*CDKN1B)*, which are associated with the hyperparathyroidism-jaw tumor syndrome, familial hypocalciuric hypercalcemia type 1, and MEN4, respectively. Here, we report a heterozygous c.1138C>T (p.Leu380Phe) *CDC73* germline variant in a clinically diagnosed MEN1 patient, based on combined occurrence of primary hyperparathyroidism, acromegaly, and a PNEN. Characterization of the PNEN confirmed it was a neuroendocrine neoplasm as it immuno-stained positively for chromogranin and glucagon. The rare variant p.Leu380Phe occurred in a highly conserved residue, and further analysis using RNA-Scope indicated that it was associated with a significant reduction in *CDC73* expression in the PNEN. Previously, *CDC73* mutations have been reported to be associated with tumors of the parathyroids, kidneys, uterus, and exocrine pancreas. Thus, our report of a patient with PNEN and somatotrophinoma who had a *CDC73* variant, provides further evidence that *CDC73* variants may result in a MEN1 phenocopy.

Multiple endocrine neoplasia type 1 (MEN1) is characterized by the combined occurrence of parathyroid tumors, pancreatic neuroendocrine neoplasms (PNENs), and pituitary adenomas [1]. In addition, some MEN1 patients may develop carcinoid tumors of the thymus, bronchus, and gut, and women may have an increased occurrence of breast cancer [1, 2]. A patient may be considered to have MEN1 on the basis of clinical, familial, and genetic criteria [3-7]. A clinical diagnosis is based on the combined occurrence of 2 or more MEN1-associated tumors in a patient; familial MEN1 is diagnosed upon occurrence of one of the MEN1-associated tumors, in a first-degree relative of a patient with a clinical diagnosis of MEN1; and a genetic diagnosis of MEN1 is established on identification of a germline *MEN1* mutation in an individual who does not have clinical or biochemical manifestations of MEN1 [3-7]. In > 75% of patients, MEN1 is caused by mutations, including whole or partial gene deletions, in-frame and frameshift deletions or insertions, splice site, nonsense and missense mutations, of the tumor suppressor gene *MEN1*, which encodes the protein menin [8, 9]. The remaining < 25% of patients, may have mutations in as-yet unidentified genes, or may represent phenocopies [3, 10]. Phenocopies, which refer to genetic disease manifestations without the specific gene mutation, can confound clinical diagnosis of hereditary disorders and therefore have implications for the management and treatment of both the patient and their relatives [3, 10]. Furthermore, it has been reported that mutations in genes, for example, the cell cycle division 73 (*CDC73*), calcium sensing receptor (*CASR*), and cyclin dependent kinase inhibitor 1B (*CDKN1B*), can result in MEN1 phenocopies [3, 10]. In this study, we present the clinical details of a patient with mild primary hyperparathyroidism, acromegaly, and a PNEN, who did not have a *MEN1* mutation, but instead had a *CDC73* germline variant that resulted in reduced *CDC73* expression in the PNEN, thereby revealing a possible role for *CDC73* in the etiology of PNENs.

## Case Presentation

The patient was diagnosed with acromegaly at age 22 years. He had transsphenoidal surgeries at age 22 and 28 years, with radiation at age 23 years, followed by long-term medical therapy with somatostatin analogs initially, until he developed hyperglycemia and was given the growth hormone receptor antagonist, pegvisomant for approximately 10 years. At the last follow-up at age 74 years, he had a normal insulin-like growth factor 1 of 110 (34-245 ng/mL) and his pituitary adenoma was stable by magnetic resonance imaging, which showed a persistently enlarged partially empty sella with minimal residual pituitary tissue. He had central hypothyroidism, adrenal insufficiency, and hypogonadism for more than 50 years as a consequence of radiation therapy and/or pituitary surgeries. He was treated with levothyroxine 250 mcg daily, prednisone 10 to 12 mg daily because he was unable to tolerate physiologic dosing, and testosterone gel 5 g daily.

He was observed to have mild primary hyperparathyroidism with a plasma ionized calcium concentration of 1.31 mmol/L (normal = 1.15-1.30 mmol/L), an elevated PTH concentration of 87 pg/mL (normal = 10-60 pg/mL), in the setting of normal renal function and vitamin D of 29 ng/dL (normal = 20-80 pg/dL), and a normal forearm bone mineral density (T score = 0.3). Parathyroid scintigraphy was not undertaken as surgery was not indicated. He subsequently developed vitamin D deficiency, which masked the hypercalcemia, despite an increase in the PTH concentration of 184 pg/mL. His medical history included coronary artery disease, poorly controlled hypertension, spinal stenosis, and obesity. He was wheelchair bound because of chronic back pain. His brother had hypercalcemia and kidney stones, but there was no other family history suggestive of endocrine neoplasia.

At age 70 years, during a hospitalization for a prostatic abscess, an abdominal computed tomography scan incidentally revealed a pancreatic mass. Endoscopic ultrasound with biopsy demonstrated the neoplastic cells to immunostain for synaptophysin and chromogranin A, establishing the diagnosis of a PNEN. His serum concentrations of chromogranin A, vasoactive intestinal peptide, pancreatic polypeptide, and insulin were normal, although the gastrin was slightly elevated at 178 pg/mL (normal < 100 pg/mL), which was difficult to interpret because he was taking omeprazole for gastroesophageal reflux. However, he was observed to have hyperglucagonemia (glucagon = 130 pg/mL, normal < 80 pg/mL), which may have contributed to the hyperglycemia observed with somatostatin analog treatment, but did not have weight loss, stomatitis, glossitis, or necrolytic migratory erythema, which are typical features associated with glucagonoma syndrome.

Examination upon initial presentation to Massachusetts General Hospital at approximately age 60 years revealed a height of 170 cm and weight of 108 kg. He appeared frankly acromegalic, with a prominent brow and jaw, wide tooth spacing, and large hands.

Informed written consent for DNA sequence and histological analysis was obtained from the patient with the use of protocols approved by local and national research ethics committees.

### Immunohistochemistry

PNEN serial sections (5 µm) obtained from the formalin-fixed paraffin-embedded PNEN biopsy, were dewaxed, hydrated, and stained with hematoxylin and eosin, or used for immunostaining by: antigen retrieval at 120°C in a citrate buffer solution (pH 6); blocking in 10% donkey serum; incubation with primary antibodies for insulin (AbCam (ab)7842 [11]), glucagon (ab92517 [12]), chromogranin A (ab15160 [13]), and Ki67 (ab15580 [14]), followed by appropriate horseradish peroxidase-conjugated secondary antibodies (Jackson Laboratories); and visualization using the diaminobenzidine kit (Dako), as previously described [15].

**Figure 1.**
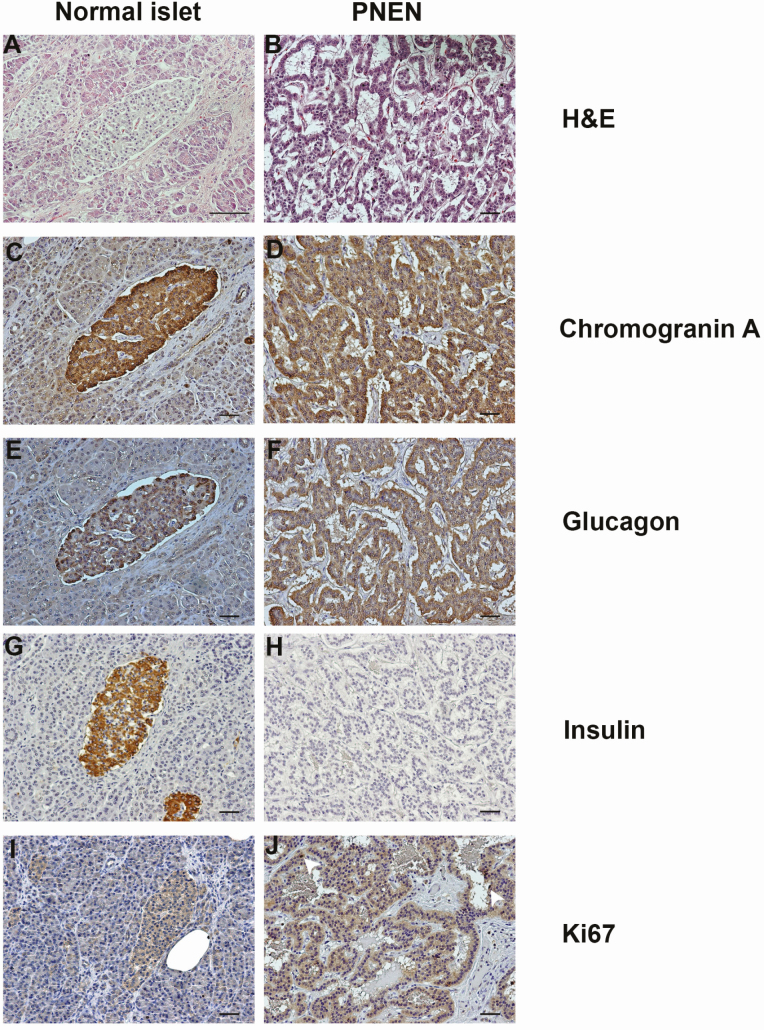
Immunohistochemical staining of a pancreatic neoplasm in patient with acromegaly, normocalcemic hyperparathyroidism and a *CDC73* c.1138C>T (Leu380Phe) variant. (A, B) Hematoxylin and eosin (H&E) staining of an adjacent normal islet and the pancreatic neoplasm. (C, D) Chromogranin A staining in an adjacent normal islet and pancreatic neoplasm. Chromogranin A expression was present in the neoplasm, confirming it was a PNEN that originated from cells of the endocrine pancreas. (E, F) Glucagon staining of an adjacent normal islet and the pancreatic neoplasm. Glucagon expression was present in the neoplastic cells indicating that the PNEN is a glucagonoma. (G, H) Insulin staining of an adjacent normal islet and the PNEN. Insulin expression was absent in the neoplastic cells. (I, J) Ki67 proliferation marker staining in a normal adjacent islet, and the neoplastic tissue. No proliferation was observed in the normal islet. A few in the neoplasm expressed Ki67, shown by white arrows, indicating that the PNEN has a low proliferation index. The scale bars represent 50 μm. PNEN, pancreatic neuroendocrine neoplasm.

Hematoxylin and eosin staining (Fig. 1A) and immunohistochemistry of the pancreatic neoplasm revealed that it immunostained for chromogranin A, compared with normal adjacent islets, thereby confirming that it was a PNEN (Fig. 1B and Fig. 1D). The neoplasm also immunostained for glucagon (Fig. 1E and Fig. 1F), but not insulin (Fig. 1G and Fig. 1H). Expression of Ki67, a marker of proliferation was not detected in normal islets (Fig. 1I), and in < 1% of neoplastic cells (Fig. 1J), and thereby indicating that the PNEN had a low proliferation rate.

### *CDC73* variant detection

**Figure 2.**
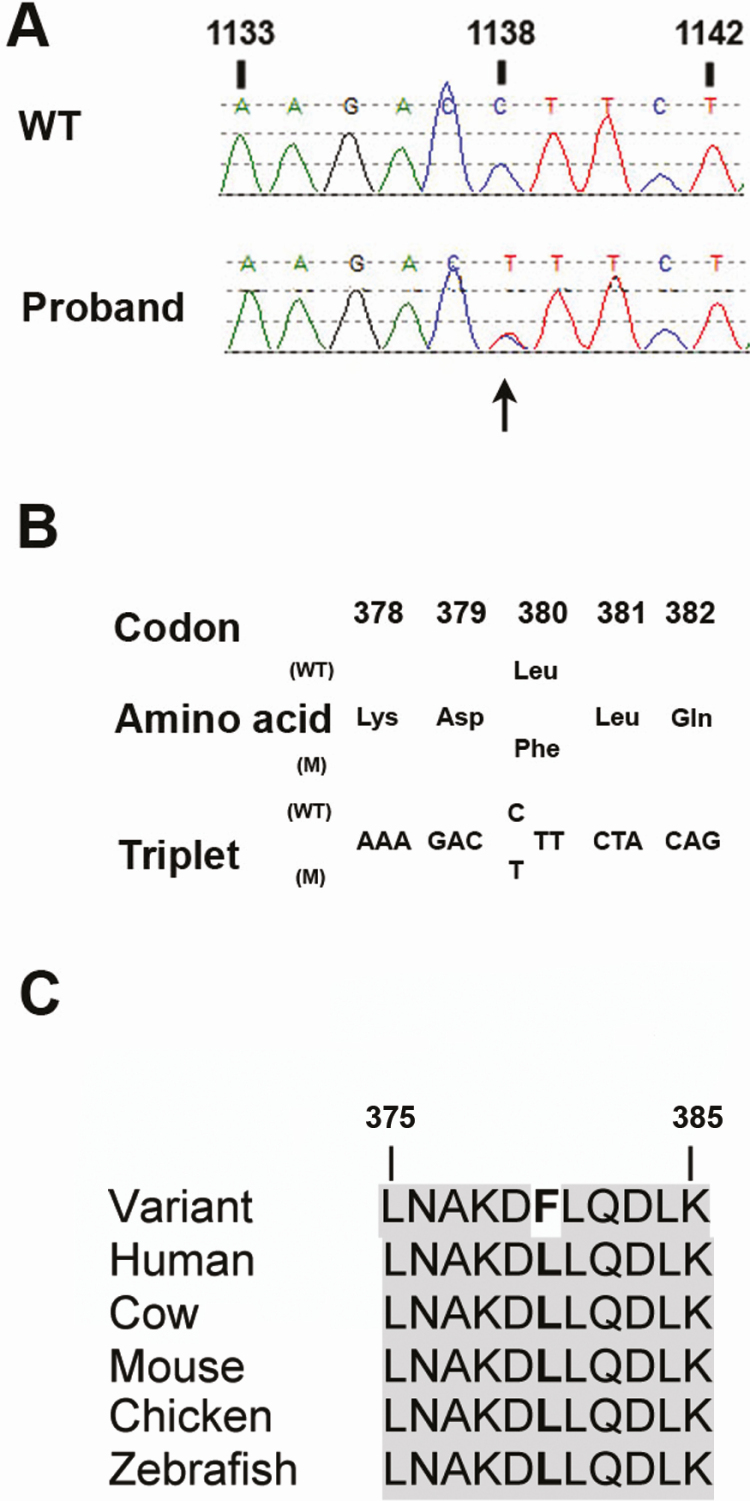
Identification of a *CDC73* Leu380Phe heterozygous missense variant in patient with acromegaly, a PNEN, and normocalcemic hyperparathyroidism. (A) DNA sequence analysis showing a heterozygous C>T transition identified in exon 13, nucleotide position c.1138, of *CDC73.* (B) The C>T transition was predicted to lead to a leucine (Leu) to phenylalanine (Phe) substitute at codon 380. (C) The leucine (L) 380 is conserved across multiple species, according to uniprot.org (November 2019). PNEN, pancreatic neuroendocrine neoplasm.

The patient was included in a study that analyzed the leukocyte DNA sequences of genes previously reported to be associated with the development of NENs, including the *CDC73, CDKN1A, CDKN1B, CDKN2B, CDKN2C*, and *AIP* genes, and a 5′ untranslated region open reading frame in *CDKN1B* (c.519-430) in which a MEN4 pathogenic mutation has been identified [16], in patients who had clinically diagnosed MEN1, but who did not have a *MEN1* mutation [17]. This revealed a heterozygous c.1138C>T transition in exon 13 of *CDC73* (Fig. 2A), that resulted in a leucine (Leu) to phenylalanine (Phe) amino acid substitution at position 380 (Leu380Phe) in the encoded protein, parafibromin (Fig. 2B) [17]. This Leu380Phe substitution represented a rare variant because it was not identified in > 125 000 sequenced alleles in The Genome Aggregation database, and the Leu380 residue is highly conserved across multiple species (Fig. 2C). Furthermore, mutation taster predicted this variant to be disease-causing (www.mutationtaster.org), and it is predicted that residues 200 through 531 of parafibromin are involved in the interaction with the polymerase-associated factor 1 (PAF1) and RNA polymerase II complex (www.uniprot.org) [18, 19]. Together, these findings indicate that the Leu380Phe alteration likely represents a pathogenic variant (mutation) rather than a benign polymorphic variant. Sufficient amounts of PNEN tissue were not available from the formalin-fixed paraffin-embedded PNEN biopsy to yield enough high-quality DNA that would allow for the conformation of the presence of this *CDC73* variant and the identification of a second mutation or loss of heterozygosity in the neoplasm.

### *CDC73* expression analysis

**Figure 3.**
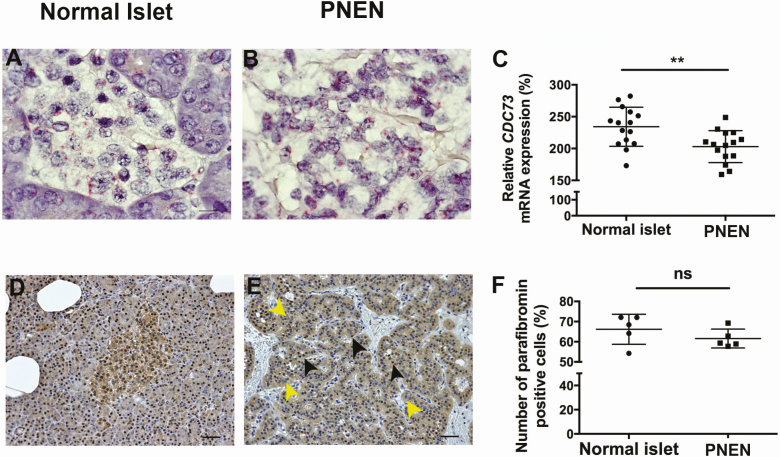
Examination of *CDC73* mRNA and parafibromin expression in a normal adjacent islet and PNEN from a patient with a *CDC73* c.1138C>T (Leu380Phe) variant. (A, B) RNA-Scope analysis of *CDC73* mRNA expression in adjacent normal islets and the PNEN. RNA expression is represented by pink dots. The scale bar represents 10 μm. (C) Quantification of *CDC73* mRNA expression in normal islets and the PNEN. Normal islets (N = 5) or PNEN fields of view (N = 5), from different sections (N = 3) were quantified. Data are represented as mean and standard deviation, **P < 0.005. (D, E) Parafibromin staining in a normal adjacent islet and PNEN. Nuclear parafibromin was seen in the majority of cells in normal islets. Nuclear parafibromin was present in some neoplastic cells (black arrows), but some neoplastic cells were negative for parafibromin expression (yellow arrows), consistent with loss of parafibromin and its role as a tumor suppressor in the etiology of neoplasms. The scale bar represents 50 μm. (F) Quantification of the number of parafibromin positive versus negative cells in normal islets and the pancreatic neoplasm that were obtained from normal islets (N = 5) and fields of view (N = 5) from the PNEN. Data are represented as mean and standard deviation. ns, not significant; PNEN, pancreatic neuroendocrine neoplasm.

To determine if the c.1138C>T (Leu380Phe) variant altered expression of *CDC73* mRNA or parafibromin protein, RNA-Scope and immunohistochemistry analysis were performed (Fig. 3). Quantification of *CDC73* and parafibromin expression was performed on neoplastic tissue (N = 5 fields of view) and adjacent normal islets (N = 5), from sections for RNA-Scope (N = 3), and immunohistochemistry (N = 1). Statistical analysis was performed using an unpaired Student *t* test, with significance at *P* < 0.05.

For RNA-Scope analysis, which can detect specific mRNA transcripts in paraffin embedded tissue sections, paraffin embedded serial PNEN sections (5 µm) were dewaxed and hydrated and RNA-Scope performed using the RNA-Scope 2.5 HD Detection Kit-RED, with a HybEZ Hybridisation System (both Advance Cell Diagnostics), and standard pretreatment and hybridization conditions, according to the manufacturer’s instructions. This revealed that normal islet cells (Fig. 3A) and PNEN cells (Fig. 3B) expressed *CDC73* mRNA. However, *CDC73* mRNA expression in PNEN cells, when compared with normal adjacent islets was significantly decreased by 13.25% (*P* < 0.005) (Fig. 3C).

To confirm if protein changes could also be observed in PNEN cells, parafibromin expression was also investigated in PNEN tissue and normal adjacent islets using immunohistochemistry, as described previously, using a parafibromin (IHC-00379, Bethyl Laboratories [20]) antibody. Nuclear parafibromin staining was seen in normal islets (Fig. 3D) and neoplastic tissue (Fig. 3E), but immunostaining was variable between individual cells within normal islets and PNEN tissue, with expression ranging from 0 to low and high intensity (Fig. 3D and 3E). Overall expression of parafibromin in PNEN cells and normal islets was not significantly different (*P* = 0.277) (Fig. 3F), with 61% and 66% of PNEN cells and cells in normal adjacent islets, respectively immunostaining for parafibromin.

## Discussion

Our results, which expand the spectrum of tumors associated with *CDC73* germline variants to potentially include somatotrophinoma and PNENs, provide further evidence that *CDC73* variants may result in a phenocopy of MEN1. Furthermore, the PNEN containing glucagon, and hyperglucagonemia in the patient reported in this study, were not associated with the clinical features of the glucagonoma syndrome, and this is consistent with the findings reported in > 80% of MEN1 patients with glucagonomas [21-23].

*CDC73* germline mutations are usually associated with occurrence of the hyperparathyroidism-jaw tumor (HPT-JT) syndrome [24, 25], which is an autosomal dominant disorder characterized by the occurrence of parathyroid tumors that are often carcinomas, and ossifying fibromas of the jaw [25-28]. HPT-JT patients also frequently develop uterine and renal tumors, and on rare occasions tumors of the thyroid, testis, and pituitary lactotrophs [27-29]. A tumor of the exocrine pancreas (a pancreatic ductal carcinoma) has also been reported in an HPT-JT patient [27]. However, 30% of *CDC73* mutation carriers exhibit nonpenetrance, and thus the lack of HPT-JT-associated tumors in our patient with the *CDC73* Leu380Phe variant is not unusual [30]. *CDC73* germline mutations are also associated with occurrence of nonsyndromic parathyroid carcinomas, but have previously not been reported to be associated with somatotrophinomas or PNENs. Furthermore, *CDC73* somatic mutations are also reported to occur in nonsyndromic parathyroid carcinomas [25, 31, 32]. Moreover, somatic *CDC73* variants that are likely pathogenic are reported to occur in > 1% of 460 PNENs (Table 1), but not in any of 85 pituitary adenomas, which included 31 somatotrophinomas [33], thereby indicating a possible role for *CDC73* abnormalities in PNEN development.

**Table 1.** *CDC73* (Transcript ENST000003674355) Single Nucleotide Variants (SNVs) Reported in 8 of 460 PNENs of Unspecified Subtypes

Coding Change	Protein Change	FATHMM^*a*^ Score	No. of Times Observed
c.737A>G	p.Glu246Gly	0.91	1
c.991G>A	p.Glu331Lys	0.92	1
c.229C>G	p.Arg77Gly	0.98	1
c.547G>C	p.Ala183Pro	0.99	1
c.556A>G	p.Lys186Glu	0.99	1
c.883C>G	p.Gln295Glu	0.98	1
c.1353A>G	p.Ala451=	0.05	2

Identification of the SNVs was undertaken by analyzing multiple datasets publically available in the COSMIC Database [33].

^*a*^FATHMM—Functional Analysis through Hidden Markov Models is a prediction tool for the functional consequences of a single nucleotide variant, a score of > 0.7 is defined as pathogenic.

*CDC73* encodes the protein parafibromin, which is part of the RNA polymerase II/PafI complex that is important for gene transcription, and it functions as a tumor suppressor through regulation of genes including the cell cycle regulator cyclin D1, and the loss of *CDC73* expression is expected to be associated with development of tumors [19, 24, 34]. To investigate the causative role of the c.1138C>T (Leu380Phe) *CDC73* variant, which involves a rare and a conserved residue, in the etiology of the PNEN, we used RNA-Scope analysis and showed a significant decrease in *CDC73* mRNA expression in the PNEN, when compared with normal adjacent islets (Fig. 3C). However, a decreased expression of parafibromin could not be detected by immunohistochemistry, in the PNEN, when compared with normal islets (Fig. 3F). Explanations for this finding include: disruption of the function of the transcription complex, without alteration of protein expression by the Leu380Phe variant, which is predicted to be in the binding region of parafibromin with RNA polymerase II/PafI complex [35]; an altered turnover of the protein resulting from the variant increasing the stability of parafibromin; the parafibromin protein forming complexes with additional proteins [19, 36]; or a reduced sensitivity of immunohistochemistry compared to RNA-Scope.

The findings of a MEN1 phenocopy resulting from a *CDC73* variant, which has been previously reported in only 1 patient with primary hyperparathyroidism and a prolactinoma, and whose family had HPT-JT [3], raise the possibility that parafibromin and menin may share a common pathway. This is plausible because parafibromin is part of the PafI complex, which is essential for the establishment of histone modifications during transcriptional elongation, and menin has been reported to associate with a SET-like histone methyl transferase complex that methylates histones [19, 36, 37]. Thus, altered functions in the downstream epigenetic pathways may provide a basis for the similarities in tumor development. Furthermore, another Paf complex member, novel pancreatic differentiation 2, is an oncogene associated with the development of PDAC [38]. Therefore, it is possible that, similar to the mechanism by which RASSF1 and menin cooperatively modulate KRAS signalling [39], parafibromin and menin can both regulate the activity of Paf complexes in regulating gene transcription, either directly or through epigenetic mechanisms.

The identification of phenocopies also has clinical implications for the patients and their relatives because of the risks of developing the different tumors that are associated with each syndrome are different. Thus, the risks of parathyroid carcinomas, uterine tumors, and renal tumors are < 1% in MEN1 patients but much higher at ~15%, ~75%, and ~15%, respectively, in HPT-JT patients [1, 5, 25]. However, it is important to note that > 80% of MEN1 patients with a PNEN that immunostain for glucagon will not have the characteristic clinical features of glucagonoma syndrome [22]. In contrast, the risks of developing PNENs, pituitary adenomas, adrenal tumors, and carcinoids are < 1% in HPT-JT patients, but are much higher at 50% to 70%, 20% to 40%, 20% to 40%, and 2% to 10%, respectively, in MEN1 patients [1, 5, 25]. Finally, our findings indicate that patients with glucagonomas and other MEN1-associated tumors who do not have *MEN1* mutations [22] should be offered testing for *CDC73* variants.

Thus, our results potentially expand the spectrum of tumors associated with *CDC73* variants to include somatotrophinomas and PNENs, and provide further evidence that *CDC73* variants may result in a phenocopy of MEN1.

## Data Availability

All data generated or analyzed during this study are included in this published article or in the data repositories listed in References.

## Abbreviations

CASR: calcium sensing receptor
CDC73: cell cycle division 73
CDKN: cyclin-dependent kinase inhibitor
HPT-JT: hyperparathyroidism-jaw tumour syndrome
MEN1: multiple endocrine neoplasia type 1
PNENs: pancreatic neuroendocrine neoplasms
PAF1: polymerase-associated factor 1

## Additional Information

***Declaration of Interest:*** L.B.N. has served as a consultant for Ipsen and Pfizer and has received research funds from Ipsen and Chiasma; her conflicts were reviewed and are managed by Massachusetts General Hospital and Partners HealthCare in accordance with their conflict of interest policies. The other authors have no financial interests to declare.
